# Structurally-constrained optical-flow-guided adversarial generation of synthetic CT for MR-only radiotherapy treatment planning

**DOI:** 10.1038/s41598-022-18256-y

**Published:** 2022-09-01

**Authors:** Rajat Vajpayee, Vismay Agrawal, Ganapathy Krishnamurthi

**Affiliations:** 1 Department of Engineering Design, Indian Institute of Technology Madras, Chennai, India; 2 Robert Bosch Center for Data Science and Artificial Intelligence, Indian Institute of Technology Madras, Chennai, India

**Keywords:** Image processing, Machine learning, Computer science

## Abstract

The rapid progress in image-to-image translation methods using deep neural networks has led to advancements in the generation of synthetic CT (sCT) in MR-only radiotherapy workflow. Replacement of CT with MR reduces unnecessary radiation exposure, financial cost and enables more accurate delineation of organs at risk. Previous generative adversarial networks (GANs) have been oriented towards MR to sCT generation. In this work, we have implemented multiple augmented cycle consistent GANs. The augmentation involves structural information constraint (StructCGAN), optical flow consistency constraint (FlowCGAN) and the combination of both the conditions (SFCGAN). The networks were trained and tested on a publicly available Gold Atlas project dataset, consisting of T2-weighted MR and CT volumes of 19 subjects from 3 different sites. The network was tested on 8 volumes acquired from the third site with a different scanner to assess the generalizability of the network on multicenter data. The results indicate that all the networks are robust to scanner variations. The best model, SFCGAN achieved an average ME of 0.9   5.9 HU, an average MAE of 40.4   4.7 HU and 57.2   1.4 dB PSNR outperforming previous research works. Moreover, the optical flow constraint between consecutive frames preserves the consistency across all views compared to 2D image-to-image translation methods. SFCGAN exploits the features of both StructCGAN and FlowCGAN by delivering structurally robust and 3D consistent sCT images. The research work serves as a benchmark for further research in MR-only radiotherapy.

## Introduction

Magnetic Resonance (MR) imaging and Computed Tomography (CT) are two widely used methods for radiotherapy planning and have been proved to be effective diagnosis methods. Over the last decade, the use of MR imaging to support radiation therapy (RT) treatment planning has considerably increased, as MR provides high contrast of soft tissues compared to CT images, enabling better delineation of organs at risk (OAR) Schmidt and Payne^23^, Njeh^20^, Devic^7^. Moreover, MR imaging offers more information about lesion activities, such as cellular density and functional imaging of lesions. The limitation of MR in RT planning is its inability to provide the electron density information needed for dose calculation. In contrast, CT imaging provides electron density information, an essential component in dose calculation Van der Heide et al.^26^, Kazemifar et al.^14^, Ulin et al.^25^. Since there is no direct relationship between electron density information and MR intensity values, it is prevalent to find a combined pipeline that includes MR imaging for precise delineation of OARs and CT simulation for dose calculation. Involving CT acquisition in the workflow has drawbacks like increased radiation exposure and financial cost. In an attempt to reduce the radiation exposure to patients, Wang et al.^27^ have introduced a method to utilize the merits of ATV (Anisotropic Total Variation) and reweighted technique to achieve better limited-angle CT reconstruction. Chen et al.^4^ have also proposed an approach to enhance edge information in CBCT reconstruction, which uses information obtained by mapping edge contours from existing CT to CBCT. Moreover, the delineations on MR images had to be translated to CT domain using image registration, which introduces spatial uncertainties, and is considered the weakest link in the radiotherapy workflow Njeh^20^. To overcome these issues, several methods have been developed for the generation of synthetic CT (sCT) from MR images, which can be coarsely divided into 4 categories, (1) Atlas-based methods, (2) Bulk density methods, (3) Probabilistic based methods and (4) machine learning methods Largent et al.^16^.

Hofmann et al.^13^ was the first atlas-based approach for the computational conversion of MR to CT, achieving an MAE of 100.7 Hounsfield Units (HU). The bulk density method was initially introduced as a method for sCT generation. Anatomical contours from MR can be used to generate bulk anatomical density (BAD) maps Choi et al.^6^. Recent advancements in machine learning in the medical imaging field led to the development of sophisticated methods for synthetic image generation Ching et al.^5^. In 2013, Andreasen^2^ proposed an approach to investigate the use of Random Forest regression (RaFR) and Gaussian Mixture Regression (GMR) for MR to CT translation. The introduction of deep learning techniques made it possible to perform image-to-image translation generalizable across datasets. In 2014, Goodfellow et al.^10^ proposed a framework to estimate generative models using adversarial training of generative and discriminative networks. Since then, GANs have been consistently proved to be a promising path for achieving image-to-image translation tasks Alotaibi^1^. In Han^11^, a deep learning method was first introduced to generate sCT by training a generative model i.e. U-net to convert 2D MR to CT slice. They obtained an average MAE of 84.817.3 HU across test data. Maspero et al.^18^ used phase, water and fat images as input to the GAN in pelvis data and obtained an average MAE of 61.09.0 HU on test data. In Wolterink et al.^29^, a generative model was trained on an unpaired brain dataset, and Nie et al.^19^ employed a model on a paired dataset. Maspero et al.^18^ has used cGAN to obtain prostrate sCT for dosimetric calculations. So far, to our best knowledge, no deep learning based method has incorporated optical flow information for sCT generation.

This manuscript proposes deep learning models for MR to CT image generation in MR-only radiotherapy workflows for male pelvis data using a publicly available paired pelvis dataset. Test images have been taken from a scanner unseen to the trained models. This allows us to widen the scope of possibilities in training and improves the generalization of the conversion. Dosimetric (MAE and ME) and PSNR have been evaluated to quantify and compare the overall accuracy of sCT results with previously existing methods. The purpose of generating sCT from MR is to provide the electron density information for facilitating dose calculation in the absence of ground truth CT. The sCT encodes tissue information in HU which can be easily transformed to electron density in the treatment planning system (TPS) using a calibration curve. However, the dose calculation step is not incorporated in our research work, as the focus was to evaluate and compare the results among the proposed novel GANs.

## Materials and methods

### Dataset

This study was conducted on a publicly available Gold Atlas project dataset Nyholm et al.^21^, which aimed to provide means for training and validation for segmentation and sCT algorithms. The data consists of male pelvic region T1- and T2-weighted MR and CT volumes of 19 patients acquired from 3 different sites. The dataset also includes consensus delineations of nine organs for each patient based on the T2-weighted MR images. The CT volumes were deformed to fit the anatomy of MR data, enabling the use of the organ delineations on the registered CT. Table 1 provides further details of the dataset acquisition parameters.

**Table 1:** Acquisition parameters for the different sites.

		**Site 1**	**Site 2**	**Site 3**
Number of patients		8	7	4
**CT**				
	Manufacturer	Siemens	Toshiba	Siemens
	Model	Somatom Definition AS+	Aquilion	Emotion 6
	Slice thickness (mm)	3	2	2.5
	Pixel size (mm2)	0.98 0.98	1 1	0.98 0.98
	Kernel	B30f	FC17	B41s
**T2w images**				
	Manufacturer	GE	Siemens	GE
	Model	Discovery 750 w	–	Signa PET/MR
	Field strength (T)	3	1.5	3
	Sequence type	FRFSE	TSE	FRFSE
	Slice thickness (mm)	2.5	2.5	2.5
	Pixel size (mm2)	0.875 0.875	0.875 0.875–1.1 1.1	0.875 0.875
	Bandwidth (Hz/pixel)	390	200	390
	TR (ms)	6000–6600	12000-16000	9000
	TE (ms)	97	91–102	65
	Encoding direction	COL	ROW	COL

### Data preparation

To remove the background noise from the scans, all the voxels outside the body in MR and CT images were set to 0 and -1024, respectively. The pelvis axial slices were converted to 16-bit grayscale images after truncating the intensity range of the original images. Similar to Fetty et al.^9^, the upper limit to truncate the image was kept as 1400 for CT, as any values greater than 1400 were identified in rare cases, mainly in the femoral bones, which is not considered in dose planning. For MR volumes, the upper limit was restricted to 99 percentile of all the intensity values to exclude outliers, followed by min-max normalization for each volume to normalize the intensity variations in MR scans. Inter-scan differences (air pockets and structures) have not been taken into account in this study.

The first and last two axial slices were removed from the corpus to avoid the aliasing effects in MR images. All the images were resized to (256, 256) dimension using bicubic interpolation. To facilitate the direct comparison of the results, we have used the split proposed by Boni et al.^3^, i.e. site 2 and site 3 acquisitions were used as the training set, whereas site 1 was used in the testing set. Such a split also tests the robustness of the network across scanners as the testing set contains data taken from a completely different scanner. In total, there are 1015 axial slices or 11 patient volumes in the training set and 659 axial slices or 8 volumes in the test set.

### Networks

The study implements 4 different types of Generative Adversarial Networks (GANs) with a shared neural network architecture of generator and discriminator trained with different sets of loss functions, enabling the fair comparison of different loss functions to learn the given task. The first network used to obtain a baseline score in this study is the traditional CycleGAN Zhu et al.^30^. The other two networks add structural similarity (StructCGAN) and optical flow consistency (FlowCGAN) constraints during the optimization step of the generator in the GAN. The 4th network augments generator loss function with both structural similarity and optical flow constraints. All the networks were trained in a supervised fashion to establish a bidirectional mapping between CT and T2-weighted MR axial slices.

**Figure 1:**
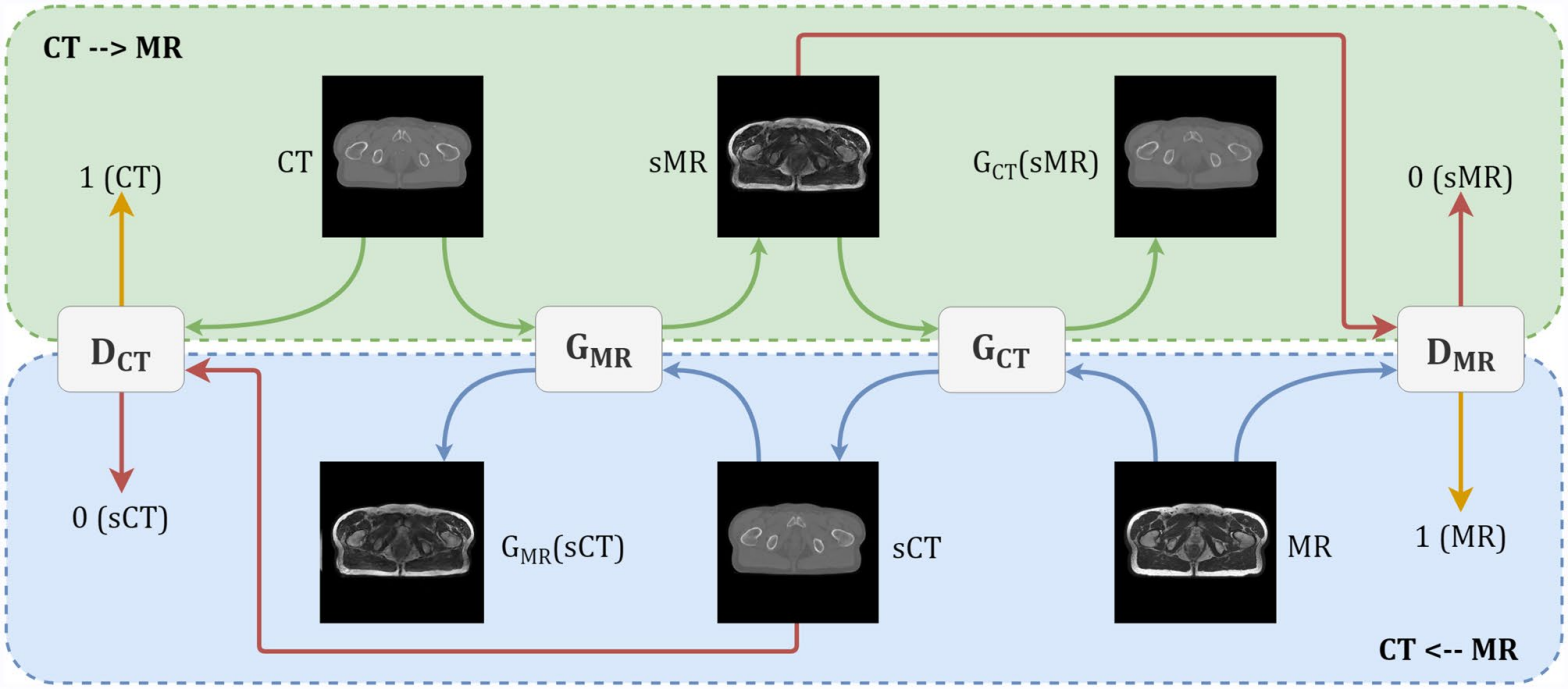
Overall pipeline for all the models. The green section shows CT to MR translation and recovery back to input CT using MR and CT generators, respectively. Similarly, the blue part corresponds to MR to synthetic CT conversion steps. If the input of the discriminator is a synthesized image, then ideally discriminator should output 0, i.e. fake image. This is denoted by the red colour arrow.

All the GANs implemented in this study have two generators and two discriminators. The generator, $G_{CT}$ translated the MR image to produce synthetic CT, i.e. $\text {sCT} = G_{{\text {CT}}}(\text {MR})$. Similarly, we have $\text {sMR} = G_{{\text {MR}}}(\text {CT})$. The two discriminators, $D_{\text {CT}}$ and $D_{\text {MR}}$ aim to distinguish between real and synthetic images. The overall pipeline common to all 4 GANs is shown in the fig. 1.

The architecture of both the generators is ResNet He et al.^12^ with 9 residual blocks. The discriminator has 5 convolutional blocks, each followed by instance normalization and ReLu non-linearity except the last layer. All ReLUs are leaky with a slope of 0.2. The first convolutional layer kernel has 64 channels, each with a size of (4, 4), and a stride of 2. Subsequently, in layer $2{\hbox {nd}}, 3{\hbox {rd}}$ and $4{\hbox {th}}$, the number of channels in the kernel keep doubling without any changes in other parameters. The final convolutional block maps its input to a single number between 0 and 1, denoting the probability of prediction as a synthetic or real image.

### Loss functions

#### Cycle-consistent GAN (CycleGAN)

Traditionally, CycleGAN training has two types of terms in loss functions. The first corresponds to adversarial losses to match the data distribution of synthetic images with ground truth images. The second is cycle consistency loss which enables the model to learn a cycle-consistent mapping. Similar to Mao et al.^17^, least-square has been used instead of negative log-likelihood objective function to improve training stability and generate higher quality images. The generator is updated by fixing the discriminator parameters and vice-versa.

The least-squares GAN loss function is provided in Eq. (1). Upon backpropagation, it minimizes the possibility of the translated image being recognized as a synthesized image by the discriminator.

1 $$\begin{aligned} \mathscr {L}_{\text {GAN}}= \mathbb {E}_{\text {CT}\sim p_{\text {data}}(\text {CT})}[(D_{\text {MR}}(\text {sMR}) - 1)^2] + \mathbb {E}_{\text {MR}\sim p_{\text {data}}(\text {MR})}[(D_{\text {CT}}(\text {sCT}) - 1)^2] \end{aligned}$$

The cycle loss function ensures the cyclic consistency between the input and generated images. The aim is to recover the input image when the synthesized image is reconstructed back to its original imaging modality. The loss function is as follows:

2 $$\begin{aligned} \mathscr {L}_{\text {cycle}} = \mathbb {E}_{\text {CT}\sim p_{\text {data}}(\text {CT})}[\ ||G_{\text {CT}}(\text {sMR}) - \text {CT})||_1\ ] + \mathbb {E}_{\text {MR}\sim p_{\text {data}}(\text {MR})} [\ ||G_{\text {MR}}(\text {sCT})) - \text {MR})||_1\ ] \end{aligned}$$

The net generator loss function is provided in Eq. (3). Here, $\lambda _{\text {cycle}}$ is taken as 10.

3 $$\begin{aligned} \mathscr {L}_{G_{\text {net}}^{\text {cycleGAN}}} = \mathscr {L}_{\text {GAN}} + \lambda _{\text {cycle}} * \mathscr {L}_{\text {cycle}} \end{aligned}$$

The discriminator loss corresponding to CT images is provided in Eq. (4). The aim is to predict label 1 for a real imaging modality and label 0 for a synthetic image.

4 $$\begin{aligned} \mathscr {L}_{D_{\text {CT}}} =&\ \mathbb {E}_{\text {MR}\sim p_{\text {data}}(\text {MR})}[(D_{\text {CT}}(\text {MR}) - 1)^2\ ] + \mathbb {E}_{\text {MR}\sim p_{\text {data}}(\text {MR})}[(D_{\text {CT}}(\text {sCT}))^2\ ] \end{aligned}$$

Similarly, the loss corresponding to $D_{\text {MR}}$ is calculated. The net discriminator loss is the average of the discriminator loss functions of individual imaging modalities, shown below.

5 $$\begin{aligned} {{\mathscr {L}_\mathscr{D}}_\text {net}} = \frac{(\mathscr {L}_{D_{\text {CT}}} + \mathscr {L}_{D_{\text {MR}}})}{2} \end{aligned}$$

The above net discriminator loss function is the same for all of our models. In contrast, the net generator loss function changes case-by-case and is explicitly defined further in the manuscript.

#### Structurally-Similar CycleGAN (StructCGAN)

MSE and L1 loss functions only measure the pixel-wise intensity difference. Structural Similarity (SSIM) index was introduced by Wang et al.^28^ to assess the perceptual image quality of a distorted image given a reference image. The comparison is performed on 3 key features: 1) luminance, 2) contrast and 3) structure. The resultant is a decimal value in the range [0, 1], wherein higher values denote greater structural similarity. The equation is defined as:

6 $$\begin{aligned} {\text {SSIM}}_{\text {CT}}&= \ \frac{(2\mu _{\text {CT}}\mu _{\text {sCT}} + C_1)(2\sigma _{\text {CT},\text {sCT}} + C_2)}{(\mu _{\text {CT}}^2 + \mu _{\text {sCT}}^2 + C_1)(\sigma _{\text {CT}}^2 + \sigma _{\text {sCT}}^2 + C_2)} \end{aligned}$$

where, $\mu _{\text {CT}}$ and $\mu _{\text {sCT}}$ represent the average of the 2D axial image slice of the reference and synthesized CT images, respectively; $\sigma _{\text {CT}}$ and $\sigma _{\text {sCT}}$ are the variance of the reference and synthesized CT image respectively; $\sigma _{\text {CT},\text {sCT}}$ is the covariance of the reference and synthesized CT image; and C1 and C2 are two hyper-parameters used to prevent unstability, and were set as 0.0001 and 0.009 respectively, to stabilize the division with the weak denominator.

Similarly, the SSIM index for MR images is also calculated. The net SSIM index is the average SSIM index of individual imaging modalities. The structural similarity loss function is constructed by subtracting the net SSIM index from 1, shown below.

7 $$\begin{aligned} \mathscr {L}_{\text {SSIM}}&= 1 - \frac{(\text {SSIM}_{\text {CT}} + \text {SSIM}_{\text {MR}})}{2} \end{aligned}$$

The authors of the SSIM index asserts that it is more practical to apply SSIM locally rather than globally. Thus, the SSIM metric was applied regionally on windows of size (11, 11), and the overall mean was taken across all the regions to backpropagate the loss function. The generator net loss is shown in Eq. (8), wherein the $\lambda _{\text {SSIM}}$ is taken as 2, and $\lambda _{\text {cycle}}$ is taken as 8.

8 $$\begin{aligned} \mathscr {L}_{G_{\text {net}}^{\text {StructCGAN}}} = \mathscr {L}_{\text {GAN}} + \lambda _{\text {cycle}} * \mathscr {L}_{\text {cycle}} + \lambda _{\text {ssim}} * \mathscr {L}_{\text {ssim} } \end{aligned}$$

#### Optical-Flow-Guided CycleGAN (FlowCGAN)

To the best of our knowledge, all the previous work in MR to sCT generation using adversarial methods used only 2D slices. Though it works quite well for individual 2D slices, there is an unavoidable discontinuity in the combined 3D volume. To facilitate interframe image consistency, we enforced L1 loss between a warped slice and a ground truth slice while training. The warping is done both forward and backward from the current slice, i.e. slices adjacent to a given slice in an epoch to enforce dual-sided optical flow consistency.

**Figure 2:**
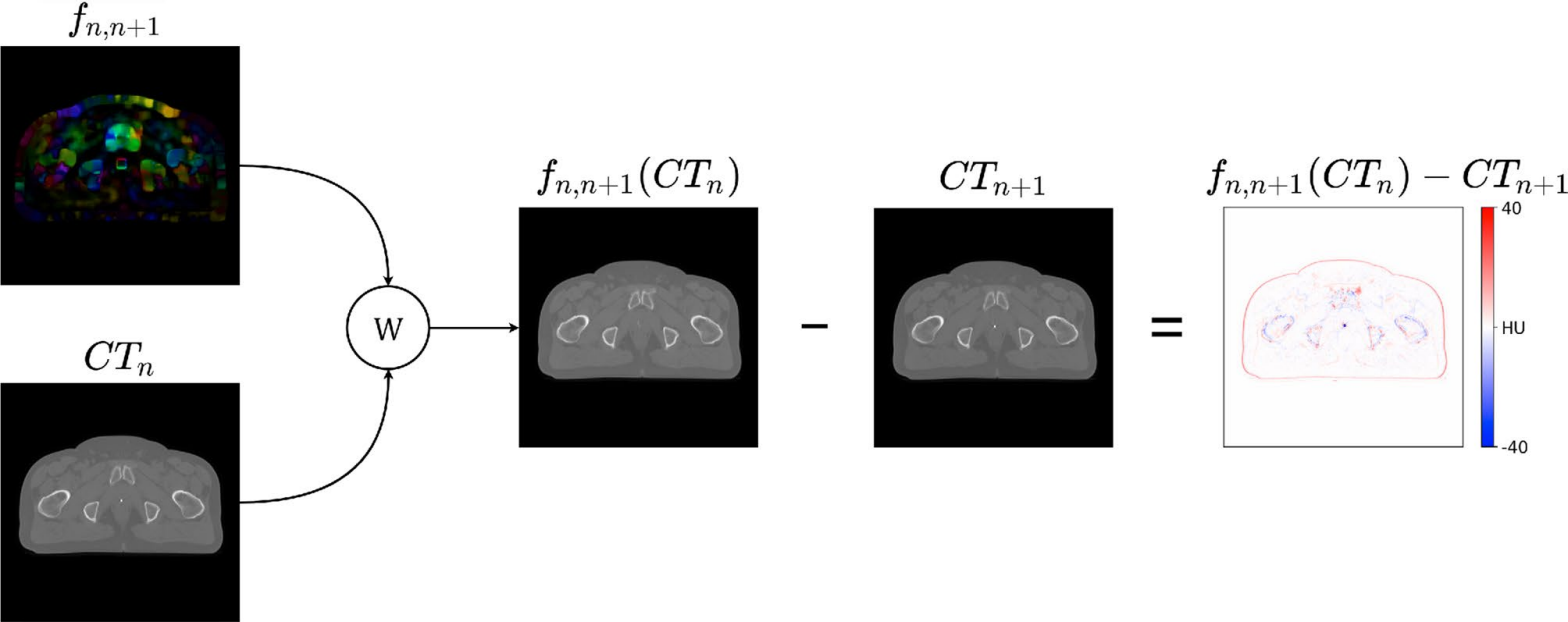
The warping of CT image from current to next slice using the flow information.

The optical flow is calculated in advance using the Farenback method Farnebäck^8^ between the ground truth pairs adjacent slices. Figure 2 shows the dense optical flow field image and the corresponding warping from the current slice to the next for original CT pairs. The Farenback method for optical flow calculation is prone to negligible error, and the same is reflected in the rightmost image of Fig. 2. The optical flow loss equation for CT is as follows:

9 $$\begin{aligned} \mathscr {L}_{\text {flow}}^{\text {CT}} =&\ \mathbb {E}_{\text {CT}\sim p_{\text {data}}(\text {CT})}[||f_{\text {n},n-1}(\text {sCT}_{n}) - \text {CT}_{n-1}||_1] + \mathbb {E}_{\text {CT}\sim p_{\text {data}}(\text {CT})}[||f_{\text {n,n+1}}(sCT_{n}) - CT_{n+1}||_1] \end{aligned}$$

Similarly, the optical flow loss for MR is also calculated. Furthermore, the optical flow loss of both the imaging modalities is averaged to obtain the net optical flow loss. The net generator loss for FlowCGAN is given in Eq. (10). Here, the $\lambda _{\text {flow}}$ is set as 2 and $\lambda _{\text {cycle}}$ as 8.

10 $$\begin{aligned} \mathscr {L}_{G_{\text {net}}^{\text {FlowCGAN}}} = \mathscr {L}_{\text {GAN}} + \lambda _{\text {cycle}} * \mathscr {L}_{\text {cycle}} + \lambda _{\text {flow}} * \mathscr {L}_{\text {flow}} \end{aligned}$$

#### Structurally-Similar Optical-Flow-Guided CycleGAN (SFCGAN)

The idea behind this network was to integrate the previous two networks so that images have high structural integrity and are coherent across slices. Therefore, both structural similarity and optical flow consistency loss were used together during the network training. The net generator loss equation is communicated in Eq. (11). The hyperparameters, $\lambda _{\text {SSIM}}$ and $\lambda _{\text {flow}}$ were taken as 2 each, and and $\lambda _{cycle}$ is taken as 6.

11 $$\begin{aligned} \mathscr {L}_{G_{\text {net}}^{\text {SFCGAN}}} = \mathscr {L}_{\text {GAN}} + \lambda _{\text {cycle}} * \mathscr {L}_{\text {cycle}} + \lambda _{\text {SSIM}} * \mathscr {L}_{\text {SSIM}} + \lambda _{\text {flow}} * \mathscr {L}_{\text {flow} } \end{aligned}$$

### Objective function

For every epoch, the generator was backpropagated followed by the discriminator. Moreover, following the strategy by Shrivastava et al.^24^ to reduce model oscillation, the discriminator was updated using the buffer of 50 previously generated images, rather than the ones produced by the latest generators. The objective function is expressed as follows:

12 $$\begin{aligned}&G_{\text {MR}}^*, G_{\text {CT}}^* \ = \ {\text {arg}} \min _{G_{\text {MR}}, G_{\text {CT}}} \ \mathscr {L}_{G_{\text {net}}}(G_{\text {MR}}, G_{\text {CT}}, D_{\text {MR}}, D_{\text {CT}}) \nonumber \\&\quad D_{\text {MR}}^*, D_{\text {CT}}^* \ = \ {\text {arg}} \min _{D_{\text {MR}}, D_{\text {CT}}} \ \mathscr {L}_{D_{\text {net}}}(G_{\text {MR}}, G_{\text {CT}}, D_{\text {MR}}, D_{\text {CT}}) \end{aligned}$$

### Training

All the networks were trained for 200 epochs with a batch size of 4. Adam optimizer Kingma and Ba^15^ was used with a learning rate of 0.0002. The learning rate was kept constant for the first 100 epochs and subsequently decayed linearly to zero over the next 100 epochs. The networks were implemented on PyTorch Paszke et al.^22^ version 1.7.1 and trained on GeForce RTX 3090 GPU. The networks used approximately 11GB of GPU RAM for training. It took around 8 hrs to train each network.

### Comparison metrics

Similar to other research work in this field Maspero et al.^18^, Fetty et al.^9^, we have estimated mean absolute error (MAE), mean error (ME) and peak signal-to-noise ratio (PSNR) between the prediction and ground truth volume inside the body contour. These parameters are calculated using these Equations:

#### MAE

13 $$\begin{aligned} \text {MAE} (\text {CT} , \text {sCT}) = \frac{1}{n}\sum _{i=1}^{n} |\text {CT(i)} - \text {sCT(i)}| \end{aligned}$$

#### ME

14 $$\begin{aligned} \text {ME} (\text {CT} , \text {sCT}) = \frac{1}{n}\sum _{i=1}^{n} (\text {CT(i)} - \text {sCT(i)}) \end{aligned}$$

#### PSNR

15 $$\begin{aligned} \text {PSNR} (\text {CT} , \text {sCT}) = \text {20log}_{10}{\frac{\text {MAX}_I}{\text {MSE}(\text {CT},\text {sCT})}} \end{aligned}$$

Where, *n* denotes the total number of pixels in a CT volume. CT(i) and sCT(i) are the intensity values (in HU) of the* i*th pixel in the ground truth and synthesized CT volumes, respectively. The $MAX_I$ value in PSNR is taken as 65535, corresponding to the maximum intensity value of a 16-bit image. MAE and ME are calculated after denormalizing the 16-bit output image to HU.

## Results

Since only the generator without backpropagation is required during the test phase, hardly 1.5GB of GPU RAM was utilized. CT synthesis took an average of 8 secs on our GPU for all the 8 test patients.

Table 2 provides the comparison metrics scores of the whole volume for all the GANs implemented in our study. The results for pix2pixHD and pix2pix taken from Boni et al.^3^ is also shown for comparison. The comparison is fair as they have also used the same dataset splits for training and testing purposes. SFCGAN outperforms all the networks with an average ME of 0.9   5.9 HU, an average MAE of 40.4   4.7 HU and 57.2   1.4 dB PSNR. The StructCGAN and FlowCGAN outperform both pix2pixHD and pix2pix network, but CycleGAN fails to exceed pix2pixHD.

All the comparison metrics were also calculated for 8 segmented organs, and the results are provided in Table 3. Comparing MAE scores, SFCGAN performs the best in the case of anal canal, penile bulb, prostate and seminal vesicles. FlowCGAN outperforms all the networks in both left and right femoral heads. For urinary bladder and rectum, pix2pixHD performs the best.

**Table 2:** The table shows the scores of different model averaged across test volumes inside the whole body contour. *Taken from Boni et al.^3^.

Models	ME (HU)	MAE (HU)	PSNR (dB)
CycleGAN	− 9.3 4.9	49 4.4	56.1 1
StructCGAN	− 5.8 9.1	42.3 5.4	56.9 1.5
FlowCGAN	2 4.4	43.1 4.5	57 1.3
SFCGAN	**− 0.9 5.9**	**40.4 4.7**	**57.2 1.4**
pix2pixHD*	− 18.3	48.5 6	–
pix2pix*	− 11.4 19	62.0 12	–

Results from SFCGAN are in bold font.

**Table 3:** ME, MAE and PSNR values of consensus delineations of organs.

	Models	Anal canal	Penile Bulb	Prostate	Seminal vesicles	Bladder	Rectum	Femoral head L	Femoral head R
**ME (HU)**									
	CycleGAN	− 15.8 14.5	14.7 20.7	**− 1.3 24.7**	− 11 54.9	21 32.3	− 66.4 80.4	30.1 70	− 3.5 41.3
	StructCGAN	− 15.3 16.7	49.7 17.4	24.4 20.6	− 3.3 52.7	63.7 24.1	− 104.7 86.2	28 64.1	24 39.5
	FlowCGAN	− 8.9 25.3	32.8 14.1	18.1 17.1	− 13.2 59.9	65.9 19.8	− 79.2 88.7	14.4 60.1	**− 9.5 51.2**
	SFCGAN	**− 3.8 16.8**	**4.8 17.4**	20.4 16.4	**− 1.2 32.7**	41.9 23.3	**− 63.1 90**	**12.3 58.1**	12.2 37.3
	pix2pixHD*	− 24.6 18	− 19.2 15	− 11.6 12	− 14.0 26	− 23.9 23	− 77.6 90	–	–
	pix2pix*	− 26.4 16	38.6 25	17.5 29	13.1 19	**− 0.6 31**	− 85.2 80	–	–
**MAE (HU)**									
	CycleGAN	32.5 14	25.6 15.3	52.5 19.6	67.3 32.5	61.3 20.8	119.2 58.3	111.5 32.2	104.4 15.4
	StructCGAN	23.3 12.5	53.7 13.3	48.8 10.3	53.8 26.1	80.7 18.1	119.5 80.1	90 32.4	82.1 11.2
	FlowCGAN	23.4 19.7	43.3 10.3	47 10.4	63.8 30	83.4 18.9	116.7 73.4	**89.7 24.5**	**80.6 10.6**
	SFCGAN	**21.9 10.8**	**25.4 8.5**	**44.8 11.8**	**45.9 11.4**	61.1 18.5	107.9 68.2	98.1 25.7	93.8 11
	pix2pixHD*	30.3 14	28.1 9	47.1 6	44.7 15	**49.4 12**	**101.8 78**	–	–
	pix2pix*	36.0 13	56.5 16	62.3 9	54.8 11	61.6 10	109.8 78	–	–
**PSNR (dB)**									
	CycleGAN	62.4 5.7	67.1 4.2	55.7 4.1	58.2 3.8	58.9 2.6	52.4 3.7	53.5 2.3	53.8 1.3
	StructCGAN	66.6 5.2	60.8 1.8	56.1 3.6	60.1 4.3	57.4 2	52.7 4.6	**55.3 2.6**	55.7 1.1
	FlowCGAN	66.5 6.7	62.3 1.9	56.2 3.5	58.7 4.3	56.8 1.6	52.4 4.3	**55.3 2.3**	**56.1 1.3**
	SFCGAN	**67 4.5**	**66.8 2.8**	**56.3 3.5**	**60.9 2.6**	**59.5 2.4**	**53.6 4.8**	54.5 1.9	54.9 0.8

Best results are in bold font.

**Figure 3:**
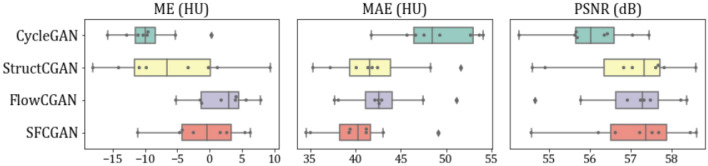
Box plots illustrating the Mean Error (ME), Mean Absolute Error (MAE) and PSNR scores distribution across test dataset volumes for CycleGAN, StructGAN, FlowCGAN and SFCGAN networks.

In Fig. 3, boxplots of ME, MAE and PSNR have shown. It can be inferred that the MAE of FlowCGAN has the minimum IQR (Inter Quartile Range) followed by SFCGAN with a slight difference of 0.52 HU. Nevertheless, SFCGAN outperforms FlowCGAN as the difference between their MAE score is 2.26 HU. It can be seen that our baseline model CycleGAN has minimum variability, followed by SFCGAN and FlowCGAN. PSNR has been computed to evaluate the quality of sCT. SFCGAN achieved the highest median of 57.33 dB, followed by FlowCGAN of 57.28 dB.

**Figure 4:**
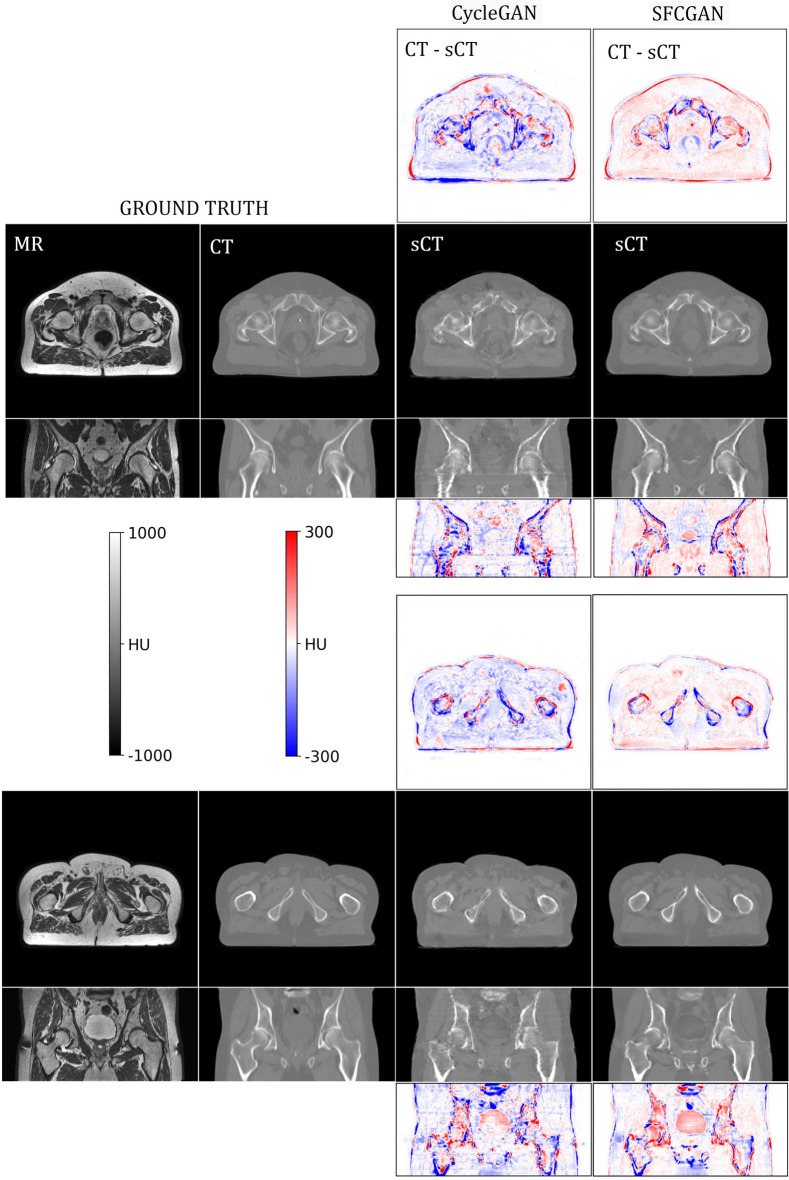
MR to CT conversion. In the above figure, column (i) represents ground truth MR (ii) represents ground truth CT . In column (iii) and (iv), the black background images are synthesized CT by CycleGAN (baseline) and SFCGAN respectively. The images in the white background represents the corresponding error in synthesized CT.

Figure 4 shows example output images of CycleGAN and SFCGAN. The coronal cross-section in the figure is obtained by stacking elements from axial slices. The axial-coronal slice pairs are taken from different patients and correspond to different pelvis segments for diverse visual inspection.

## Discussion

We inspected a new deep learning method for MR to sCT generation for dose calculation. Proposed methods consist of training 3 kinds of models (StructCGAN, FlowCGAN, SFCGAN) with CycleGAN as the base model trained with paired pelvis dataset. We evaluated (Table 2) the synthetic CT images generated by comparing their intensities with original CT using MAE and ME. The results showed that SFCGAN achieved high compliance between the HU maps for both CT images by showing the lowest ME and MAE of -0.9   5.9 and 40.4   4.7 respectively. Also, PSNR has been used to evaluate the quality of reconstructed images and SFCGAN achieved the highest 57.2   1.4, which is superior to our baseline model CycleGAN.

Figure 4 shows the output generated by SFCGAN. It can be seen that differences in the intensity values of soft tissues in original CT and sCT is minimal. Although sCT has brighter pixels at the edges compared to the CT, there is a considerable difference in intensity values at the bone and soft tissue boundaries. Additionally, incorporating optical flow information as an input enables our model to maintain the interframe consistency in sCT. The interframe consistency is reflected in the continuity of bones and tissues along the vertical axis in the coronal cross-section of SFCGAN results. Unsurprisingly, the same is absent in CycleGAN.

The study can be further improved by including a larger dataset. Unfortunately, to the best of our knowledge, there is no alternative publicly available paired pelvis dataset for research purposes. To minimize the limitation of the study posed by small dataset, the testing is performed on 8 pelvis volumes taken from a completely different site. The results are promising across unseen scanner data and indicate generalizability of the network across multiple scanners. These results show that our proposed model provides an accurate and reproducible sCT which can improve the robustness of MR-only radiotherapy workflow and reduce the clinical workload of performing a CT scan for the patients who are unable to undergo a CT scan due to radiation exposure.

## Conclusion

In this work, we developed different types of CycleGAN models for generating synthetic medical images and compared the results against state-of-the-art results Boni et al.^3^. FlowCGAN can serve as an approximation to provide the accurate motion of pixel values in sCT images to maintain interframe consistency. StructCGAN generates CT images that are structurally robust and can provide better accuracy for diagnosing fractures. SFCGAN exploits the features of both StructCGAN and FlowCGAN by proving structurally robust and frame consistency in the sCT images. The model performed well on the dataset acquired from entirely different sites, showing the model’s generalizability. The model serves as a benchmark for further research in MR-only radiotherapy and dose estimation in pelvis scans.

## Data Availability

The data that support the findings of this study are available from Tufve (record owner), but restrictions apply to the availability of these data, which were used under license for the current study. Data are however available from the owner upon reasonable request at https://zenodo.org/record/583096 We hereby confirm that all methods were carried out in accordance with relevant guidelines and regulations as publicly available data is used in the study.
